# Differences in Medical Expenditures for Men and Women with Diabetes in the Medical Expenditure Panel Survey, 2008–2016

**DOI:** 10.1089/whr.2020.0050

**Published:** 2020-09-15

**Authors:** Joni S. Williams, Leonard E. Egede

**Affiliations:** ^1^ Division of General Internal Medicine, Department of Medicine, Medical College of Wisconsin, Milwaukee, Wisconsin, USA.; ^2^ Center for Advancing Population Science (CAPS), Medical College of Wisconsin, Milwaukee, Wisconsin, USA.

**Keywords:** adults, diabetes, disability, functional limitation, medical expenditures, sex differences

## Abstract

***Background:*** Evidence suggests that women have increased health care costs; however, little is known about expenditures for women with diabetes compared with men with diabetes. The objective of this study was to calculate expenditures for men and women and to identify factors associated with increased costs in women.

***Materials and Methods:*** Adults with diabetes (*n* = 2,078) from the 2011 Medical Expenditure Panel Survey (MEPS) were identified. A generalized linear model with gamma distribution and log link was used to estimate incremental expenditure in women compared with men and to identify reasons for this difference. Sequential models were analyzed by entering variables in blocks (demographics, medical comorbidities, mental comorbidity and disability, and functional limitation). IRB approval was waived for this secondary data analysis.

***Results:*** Unadjusted mean total expenditures were $12,485 for women with diabetes compared with $10,828 for men (*p* = 0.04). In the model with demographic variables and medical comorbidities, expenditures for women increased to $1,720 (*p* = 0.03) (95% confidence interval [CI] 164–3,266) compared with men. With a comorbid mental health disorder, expenditures for women decreased slightly, but they remained significantly higher than for men at $1,668 (*p* = 0.04) (95% CI 104–3,222). In the final analysis with all variables, incremental expenditures increased by $1,314 for women compared with men and were no longer statistically significantly higher than for men (*p* = 0.10; 95% CI −257 to 2,933).

***Conclusions:*** Our findings show that women with diabetes have increased expenditures for health care compared with men with diabetes. Increased functional limitation and disability in women account for incremental increases in costs, which suggest a need for more efforts to manage disability burden in women with diabetes.

## Introduction

Diabetes is a chronic disease that accounts for a high financial burden, increased utilization of health care resources, and significant lost productivity.^1–3^ More than 20% of total health care expenses in the United States is for people with diagnosed diabetes, of which type 2 diabetes accounts for 90%–95% of cases.^1^ More than half (53%) of the medical costs associated with diabetes are secondary to managing and treating diabetes-related complications such as coronary heart disease and stroke,^4,5^ which account for ∼57% of these direct costs overtime.^6^ By the year 2034, individuals with diagnosed diabetes is expected to double, with total medical expenditures tripling to $336 billion, an estimate nearly reached in 2017.^6,7^

Individuals with diagnosed diabetes incur average medical costs higher than $16,752 annually (with more than $9,600 being due to diabetes) and have medical expenditures 2.3 times higher than that of people without diagnosed diabetes.^3^ In 2012, diagnosed diabetes was estimated to total ∼$245 billion in costs, representing a 41% increase in total expenditures within 5 years according to the American Diabetes Association.^7^ In 2017, the cost of diagnosed diabetes rose to ∼$327 billion—a 26% increase over 5 years—accounting for $237 billion in direct medical expenditures and $90 billion in reduced productivity.^3^

Direct costs associated with diabetes are due to inpatient hospitalizations (43%), prescription medications for comorbid conditions and diabetes-related complications (18%), diabetes medications and supplies (12%), physician office-based visits (9%), and stays at skilled nursing and residential care facilities (8%).^3,7–9^ In addition to reduced productivity for both the employed and unemployed with diabetes, increased absenteeism, disease-related disability, restricted activity, and early mortality account for increased indirect costs in diagnosed diabetes.^3,7,10^

In general, evidence suggests that women have increased health care costs compared with men as a result of increased utilization, longer life expectancies, and reproductive conditions, including gynecologic issues, pregnancy, and childbirth.^11–14^ Despite fewer complications in women than men, there is also speculation that women with diabetes have higher total medical expenditures than men with diabetes.^7^ In addition, previous studies have shown certain factors, including female sex, to increase the risk of functional disability,^15–19^ which may account for increased expenditures in women with diabetes.

More than 13 million women in the United States currently have diabetes,^10^ and these estimates are projected to increase with diabetes diagnoses in women comprising most cases by 2050,^20^ resulting in higher overall medical costs for women overtime. To date, the evidence about expenditures for women with diabetes compared with men with diabetes is limited. Therefore, the objective of this study was (1) to calculate expenditures among a nationally representative sample of men and women with diabetes, hypothesizing that women would have higher costs, and (2) to identify factors associated with increased costs in women.

## Materials and Methods

This study used data from the 2011 Medical Expenditures Panel Survey (MEPS) to estimate expenditures by sex and identify underlying factors associated with increased costs among adult respondents (≥18 years of age) with diabetes. IRB approval was waived for this secondary data analysis.

### Data source

The MEPS is a large survey of families and individuals, medical providers, and employers within the United States that is cosponsored by the Agency for Healthcare Research and Quality (AHRQ).^21–23^ It provides nationally representative estimates of health care services, frequency of utilization, and expenditures. Analyses were performed by using data from adult respondents to the MEPS Household Component (HC) who self-reported a diagnosis of diabetes (either type 1 or type 2 or gestational diabetes, as MEPS does not stratify according to type).

An MEPS family is defined as (1) two or more people living together in the same household, who are related by blood, marriage, adoption, or foster care; (2) unmarried individuals who live together and consider themselves a family unit; or (3) individuals who are single (*i.e.*, live without a relative or significant other).^21^ In the HC, self-reported, detailed information is collected for each person within the household, including those who are not present during the interview (*i.e.*, college students), on demographic characteristics, health conditions, health status, medical services use, charges and source of payments, access to care, health insurance coverage, income, and employment.^21^

### Sample characteristics and covariates

Demographic variables used for this study included age, race/ethnicity, marital status, education, insurance, total annual family income, metropolitan statistical area (MSA), region, comorbidity status (medical and psychological), activities of daily living (ADLs) including instrumental activities, and physical functioning limitations.

Age was divided into four categories: 18–24, 25–44, 45–64, and 65 years and older. Race/ethnicity was categorized as non-Hispanic white (NHW), non-Hispanic black (NHB), Hispanic, and Other. Marital status was defined as currently married, widowed/divorced/separated, or not/never married. Education was reported as (1) less than high school graduate, (2) high school graduate or general equivalency diploma, (3) some college or college graduate, and (4) graduate school attendee or graduate. Insurance levels were classified as (1) private, (2) public, or (3) uninsured. Total annual family income as a percentage of the poverty line was defined as (1) poor/negative (less than or equal to the poverty line and negative income), (2) near poor (<125% of the poverty line), (3) low income (125% to <200% of the poverty line), (4) middle income (200% to <400% of the poverty line), or (5) high income (≥400% of the poverty line). The MSA was listed as non-MSA or MSA. The regions of residence were divided into the (1) Northeast, (2) Midwest, (3) South, and (4) West.

### Comorbidity burden

The MEPS designated certain conditions as priority conditions due to their relatively high prevalence and because generally accepted standards for appropriate clinical care have been developed. The following medical conditions were included in the analyses for this study: hypertension, cardiovascular disease, stroke, high cholesterol, emphysema, asthma, joint pain, and arthritis. Depression was included as the mental health comorbidity.

Depression was based on self-report by using the Patient Health Questionnaire-2, which assessed the frequency of individuals' depressed mood and decreased interest in usual activities over the past 2 weeks.^24^ Respondents were asked whether they had little interest or pleasure or felt down/depressed/hopeless. The scores of the two items were summed (answer choices ranged from 0 to 3), with total scores ranging from 0 through 6, where higher scores indicate a tendency toward depression.^24^ Using a cut-point of ≥3 has sensitivity and specificity of 83% and 92%, respectively, for identifying major depression and is used frequently for screening of depressive symptoms and to indicate the probability of depression and rarely to measure severity or a clinical diagnosis of depression.^24^

### Functional limitation variables

Three variables from the MEPS were used to measure functional limitation: ADL, instrumental activities of daily living (IADL), and physical functioning. Activities performed daily to care for self are called ADLs and include walking, bathing, dressing/grooming, toileting, brushing teeth, and eating/feeding. If assistance was needed to conduct any of these functions, individuals were designated as having limited ability to perform ADLs. Similarly, individuals were asked whether they received help or required supervision with more complex daily activities such as cooking/preparing meals, driving, managing medications, paying bills, doing laundry, and using technology (*i.e.*, telephone or computer) to assess limitations with IADLs.^22^ To assess functional limitation, individuals were asked the following question: “Does anyone in the family have difficulties walking, climbing stairs, grasping objects, reaching overhead, lifting, bending or stooping, or standing for long periods of time?”^21^

### Predictor variable

The primary independent variable was sex and was self-reported. This information was verified during each interview.^21^ According to the MEPS HC Consolidated Data File and codebook, sex was determined based on the respondent's first name, and second, through family relationships if sex of the respondent was unverifiable, unavailable, or unknown, it. If the participant's first name or family relationship did not indicate the sex, it was assigned randomly as a last resort; this was the case for five of the participants.^21^

### Outcome variable

The primary dependent variable was the direct cost calculated as total medical expenditure, or what was paid for health care services, which included out-of-pocket expenses and payments by insurance (private, Medicare, and Medicaid) and other sources.^21^ In addition, total medical expenditures include office-based medical care, outpatient visits, emergency room visits, inpatient hospitalizations (including zero-night stays), prescription medications, dental care, home-based health care, and other medical expenditures.^21^ For the analyses, expenditures were adjusted to 2017 dollars by using the Consumer Price Index obtained from the Bureau of Labor Statistics.

### Statistical analyses

Three main types of analyses were performed. First, percentages calculated from chi-squared (*χ*^2^) tests summarized each demographic variable, comorbidity variable, disability variable, and functional limitation variable for the men and women in the sample (Table 1). Second, the unadjusted mean scores for total health care expenditure for men and women were calculated by using the adjusted Wald test, a parametric test useful when analyzing skewed data such as expenditure and cost data. Third, a generalized linear model (GLM) with gamma distribution and log link was used to estimate the incremental expenditures between men and women (Table 2). The Park test was used as a diagnostic test to examine the model fit. The results of the modified Park test verified the use of a gamma distribution with a log link as the best-fitting GLM to get consistent estimation of coefficients and marginal effects of medical expenditures.

**Table 1.** Sample Demographics for Men and Women with Diabetes

	All (n = 2,590)	Men (n = 1,193)	Women (n = 1,397)	p
Age (years)				0.590
18–24	0.5	0.2	0.7	
25–44	13.2	13.1	13.2	
45–64	47.3	47.8	46.8	
65+	39.1	38.9	39.2	
Race/ethnicity				<0.001^*^
Non-Hispanic White	61.6	65.7	57.6	
Non-Hispanic Black	16.2	12.8	19.5	
Non-Hispanic Other	6.7	6.3	7.2	
Hispanic	15.5	15.2	15.7	
Marital status				<0.001^*^
Married	56.2	66.7	45.6	
Widowed/divorced/separated	32.1	22.2	42.0	
Not married	11.7	11.1	12.3	
Educational level				0.392
<High school graduate	21.6	20.7	22.5	
High school graduate	33.2	33.2	33.2	
College	39.3	39.2	39.4	
Graduate school	5.9	6.9	4.9	
Insurance status				0.054
Private insurance	60.3	63.0	57.5	
Public insurance	31.2	28.5	33.9	
No insurance	8.6	8.5	8.7	
Annual family income level				<0.001^*^
Poor/negative income	15.6	13.3	17.8	
Near poor	5.1	3.6	6.7	
Low income	15.0	12.4	17.7	
Middle income	31.6	32.0	31.3	
High income	32.6	38.7	26.6	
MSA				0.300
MSA	18.5	19.6	17.4	
Non-MSA	81.5	80.4	82.6	
Region				0.919
Northeast	15.8	15.5	16.0	
Midwest	23.2	23.5	23.0	
South	42.2	42.7	41.6	
West	18.9	18.3	19.4	
Hypertension				0.287
No	23.6	22.3	24.9	
Yes	76.4	77.8	75.1	
Cardiovascular disease				0.046^*^
No	68.6	66.0	71.2	
Yes	31.4	34.0	28.8	
Stroke				0.668
No	89.4	89.7	89.0	
Yes	10.7	10.3	11.0	
High cholesterol				0.973
No	28.5	28.5	28.6	
Yes	71.5	71.5	71.4	
Emphysema				0.705
No	96.0	95.8	96.2	
Yes	4.0	4.2	3.8	
Asthma				<0.001^*^
No	86.8	90.7	82.8	
Yes	13.2	9.3	17.2	
Joint pain				0.005^*^
No	46.1	50.2	42.1	
Yes	53.9	49.8	57.9	
Arthritis				<0.001^*^
No	50.4	58.0	42.8	
Yes	49.6	42.0	57.2	
Depression				0.051
Not depressed	84.5	86.4	82.6	
Depressed	15.5	13.6	17.4	
Limited ADL				0.172
No	94.9	95.7	94.1	
Yes	5.1	4.3	5.9	
Limited IADL				0.420
No	91.8	92.4	91.2	
Yes	8.2	7.6	8.8	
Limited physical functioning				<0.001^*^
No	68.5	73.5	63.4	
Yes	31.5	26.5	36.6	

All numbers represent percentages.

^*^ Statistically significant difference at *p* < 0.05.

ADL, activities of daily living; IADL, instrumental activities of daily living; MSA, metropolitan statistical area.

To identify underlying factors associated with increased costs for men and women, sequential models were computed by entering variables in blocks (sociodemographic characteristics, medical comorbidity, mental health comorbidity, disability using ADLs and IADLs, and functional limitation).

Unadjusted (Model 1) and adjusted models were computed. Adjustments were made for relevant sociodemographic characteristics, including age, race/ethnicity, marital status, education, insurance, income, MSA, region, and medical comorbid conditions (Model 2), depression (Model 3), ADLs (Model 4), IADLs (Model 5), and functional limitation (Model 6). The primary independent variable was sex, and the primary dependent variable was total medical expenditure. All variables were included in the model, because each was conceptually related to the outcome of interest. All analyses took into account the complex survey design and weighted sampling probabilities of the data source. A two-tailed alpha (*α*) of 0.05 was used to assess for significance. After estimation of each model, incremental costs were calculated by using the margins command in Stata. All analyses were performed by using Stata software version 14.0.^25^

## Results

Table 1 shows the sample demographics for men and women with diabetes. The majority of the sample (47%) was between the ages of 45 and 64 years, with those 65 years of age and older making up 39% of the sample. Nearly 62% of the sample was NHW, and 56% were married. Thirty-three percent of the sample graduated from high school, and 39% attended or graduated from college. Sixty percent had private insurance, and more than half of the sample (51%) made less than $25,000 annually. Eighty-two percent of the sample resided in a non-MSA, and the majority of the sample lived in the South (42%). Approximately 76% had hypertension, 31% had cardiovascular disease, 72% had high cholesterol, 4% had emphysema, 13% had asthma, 54% had joint pain, and 50% had arthritis. Nearly 16% reported being depressed. Approximately 5% reported limited ADL, whereas 8% reported limited instrumental ADL, and 32% reported limited physical functioning.

Forty-six percent of the sample was composed of men, with the remaining 54% being women. Approximately 42% of the women in the sample were widowed, divorced, or separated compared with 22% of men. Only 45% of the men made less than $25,000 compared with 58% of the women. More than 17% of women reported an annual family income of negative/poor or low compared with 12%–13% of men. Approximately 27% of women reported high annual family income compared with 39% of men. A significantly greater percentage of women had medical comorbidities such as asthma, joint pain, and arthritis and mental health comorbidity such as depression compared with men in the sample. A greater percentage of women were more likely to report limited ADL and limited physical functioning compared with men. Overall, statistically significant differences in the sample were observed in terms of race and ethnicity, marital status, and annual income, cardiovascular disease, asthma, joint pain, arthritis, and functional limitation.

Women had significantly higher mean expenditures totaling $12,485 compared with $10,828 for men (*p* = 0.039). These medical expenditures accounted for direct costs, which included out-of-pocket expenses and payments by insurance (private, Medicare, and Medicaid) and other sources for office-based and outpatient medical care, emergency room visits, inpatient hospitalizations, prescription medications, dental care, home-based health care, and other medical expenditures (21). Indirect costs such as absenteeism from work, reduced performance at work, reduced productivity when not in the workforce, reduced labor due to disability, and mortality (3) were not accounted for in these total expenditures.

Table 2 shows the sequential explanatory GLMs for incremental expenditures for men and women in the sample. In the unadjusted model (Model 1), women had significantly higher incremental costs compared with men at $1,657 (95% confidence interval **[**CI] 92–3,213). Significantly higher expenditures for women compared with men persisted when the model was adjusted for sociodemographic characteristics and medical comorbidities (Model 2) ($1,720; 95% CI 164–3,266). When adding depression as the mental health comorbid condition into the model (Model 3), incremental costs for women decreased, but they remained significantly higher than those for men at $1,668 (95% CI 104–3,222). As variables to assess functional limitations such as ADLs (Model 4) and IADLs (Model 5) were included in the model, incremental costs for women continued to be significantly higher and increased significantly compared with the incremental costs for men ($1,729; 95% CI 129–3,320 and $1,794; 95% CI 176–3,403, respectively). In the final analysis, when all variables, including limited physical functioning, were added into and adjusted for within the model, incremental expenditures for women continued to be higher at $1,341 compared with men (95% CI −257 to 2,933); however, the model was no longer statistically significant, indicating limited physical functioning as a factor that influences increased expenditures in women with diabetes compared with men with diabetes.

**Table 2.** Sequential Explanatory Generalized Linear Model for Incremental Expenditures for Men and Women with Diabetes

	Women versus men	
	Incremental costs	95% CI
Model 1	$1,657^*^	92 to 3,213^*^
Model 2	$1,720^*^	164 to 3,266^*^
Model 3	$1,668^*^	104 to 3,222^*^
Model 4	$1,729^*^	129 to 3,320^*^
Model 5	$1,794^*^	176 to 3,403^*^
Model 6	$1,341	−257 to 2,933

Expenditures were adjusted to 2017 dollars by using the Consumer Price Index.

^*^ Significantly significant difference at *p* < 0.05. Reference group: men.

Model 1: Unadjusted.

Model 2: Adjusted for sociodemographic characteristics + medical comorbidities.

Model 3: Adjusted for Model 2 + mental health comorbidity.

Model 4: Adjusted for Model 2 + Model 3 + ADLs.

Model 5: Adjusted for Model 2 + Model 3 + Model 4 + IADLs.

Model 6: Adjusted for Model 2 + Model 3 + Model 4 + Model 5 + physical functioning limitation.

## Discussion

In this nationally representative sample of adults with diabetes, women had significantly increased mean expenditures for health care compared with men. In unadjusted analyses, women with diabetes had higher incremental costs compared with men with diabetes. When adjusting for sociodemographic characteristics, medical and mental health comorbidities, ADLs, and IADLs in this diverse sample of adults with diabetes, women had significantly higher expenditures compared with men. However, after adjusting for physical functioning limitations, statistically significant differences in medical expenditures between the two groups no longer persisted. Although a definitive cause cannot be identified based on the design of the study or our findings, it is possible that increased physical limitation and disability might account for higher incremental costs in women with diabetes compared with men with diabetes. This finding suggests a need for more efforts to manage disability burden and improve the functional status of women with diabetes.

Our findings are supported by evidence from previous studies assessing the relationship between disability and diabetes among women. In this sample, we found increased functional limitation and disability accounted for incremental costs in women with diabetes. In a study to examine the relationship between diabetes and the incidence of functional disability in older women, Gregg et al. found the yearly incidence of any functional disability among women with diabetes to be 9.8%.^18^ In addition, they found women with diabetes to have a 42% increased risk of disability for any type of limitation in physical functioning and a risk between 53% and 98% for specific tasks that included walking two to three blocks, walking up or down stairs, doing housework, shopping, and cooking meals.^18^ Similarly, a cross-sectional analysis of 3,570 noninstitutionalized women from an urban area within the United States showed women with diabetes twice as likely to report difficulties with mobility, upper extremity functioning, IADLs or higher functioning tasks, and ADLs or self-care tasks.^20^

In addition, we found women with diabetes in this sample to have higher mean total expenditures compared with men with diabetes. Although this estimate was significantly higher for women with diabetes compared with men with diabetes, the average for both was lower than the reported average of $16,750 incurred for average medical expenditures annually for adults with diagnosed diabetes.^3^ It is important to note, however, that the mean expenditures reported in this study were based on data from adult respondents to the MEPS HC who self-reported a diagnosis of diabetes, whereas the average estimate for medical expenditure is based on a compilation of both state and national data for diabetes from multiple sources.^3^

Evidence suggests that physical disability, including limitations in mobility, ADLs, and IADLs, is a fairly common problem for individuals diagnosed with diabetes, and diabetes is a strong predisposing factor for increased risk of physical disability^15,18,26–29^; this is especially true in older adults with a long duration of diabetes, where a vast majority of research on diabetes and functional limitation has occurred.^21,30^ It is estimated that the risk of disability or limited physical functioning secondary to diabetes ranges from none to double.^26^ A systematic review and meta-analysis of the literature evaluating the relationship between diabetes and the risk of physical disability in adults found adults with diabetes to have a 50%–80% increased risk of disability compared with individuals without diabetes.^26^

In our sample, functional limitation and disability were associated with increased medical expenditure in women compared with men. Recent estimates by the American Diabetes Association show that adults with diabetes have a 3.1% higher chance of being unemployed and receiving disability payments compared with adults without diabetes.^3^ For individuals in the workforce who are unemployed, an inability to work due to diabetes-related disability is estimated at $37.5 billion in indirect costs.^3^ The resulting limitation in physical functioning for individuals with diabetes leads to a substantial impairment in quality of life for both middle- and older-aged individuals and a disproportionate burden in work disability, especially for women with diabetes.^16,27^

The exact etiology of functional disability in diabetes is not known; however, it is postulated that the cause is multifactorial and can include known complications associated with diabetes (*e.g.*, vascular and neuropathic conditions), comorbid conditions (*e.g.*, arthritis, depression, lower extremity impairment), poorly controlled diabetes (*e.g.*, hyperglycemia), and obesity.^18,19,26–31^ In addition, there has been a recent surge of evidence in the literature, suggesting a relationship between sarcopenia and diabetes.^32^ It has been shown that sarcopenia is associated with decreased metabolic rate and increased frailty (*i.e.*, decreased physical activity and weight loss with increased weakness and exhaustion).^32,33^ Diabetes is one of several chronic conditions believed to be associated with frailty, especially considering the psychological and social factors needed for adequate management. Additional research is needed to understand the relationship between disability and diabetes and the resulting impact on outcomes, especially among women who had significantly higher incremental costs compared with men in this sample.

In general, the management and treatment of diabetes and its associated complications are costly for individuals, health care systems, and the nation; a lack of insurance contributes substantially to less access to care, suboptimal medical care, poorer outcomes, and higher costs for patients with diabetes.^34^ Our findings showed that women with diabetes have higher unadjusted and adjusted incremental expenditures each year compared with men with diabetes. This is clinically significant because financially, this difference in expenditure might explain some of the observed differences in diabetes care between the two groups. Fortunately, men and women with diabetes are suited to benefit from comprehensive health care reform implemented because of the Affordable Care Act (ACA). Particularly for women, the ACA is heralded as one of the most important advances in women's health since the establishment of Medicare in 1965, as it increases a woman's access to insurance, lowers health care costs, improves the quality of health care services, and allows for cost-free preventive care.^35^

For men and women with diabetes, ACA benefits are received, generally, in terms of health insurance protections and new coverage opportunities.^36,37^ These provisions are especially important for adults with diabetes who will incur higher medical expenditures associated with diabetes and those unable to participate in the workforce secondary to diabetes-related disability. Specific examples of health insurance protections offered to patients with diabetes include: (1) guaranteed employment-based and individual coverage for diabetes without increased fees or refusal to treat (*i.e.*, nonexclusion of preexisting conditions such as diabetes); (2) coverage for young adults ≤26 years of age who are identified as dependents on their parent's insurance plan, regardless of marital status, financial independence, and living arrangements; (3) free preventative care services such as screenings for hypertension and annual wellness visits for Medicare patients; (4) no lifetime dollar limits on coverage and no annual dollar limits on essential health benefits such as prescription drugs, chronic disease management, and hospitalization; (5) plan types designed for cost-sharing between insurers and the insured; and (6) closure of the Medicare “donut hole” responsible for making it difficult for patients, especially seniors, to afford prescription medications.^36,37^

In terms of options for health care coverage provided by the ACA, patients with diabetes can (1) shop for an affordable insurance plan in the Health Insurance Marketplace, and if eligible, (2) receive financial assistance to make payments for insurance, and (3) receive Medicaid coverage if residing in a state where Medicaid expansion has been accepted,^34,36,37^ allowing for the collection and provision of state-based estimates that demonstrate the impact of diabetes-related disability on medical expenditure to more accurately inform health care policy.^38^ Provisions of the ACA should help patients with diabetes reduce expenditures associated with diabetes-related care in the long term. In addition, the ACA will improve access to care and provide a usual source of care for men and women with diabetes, which can lead to an increased use of services and better resulting outcomes. However, additional research is warranted to assess the impact of the ACA on reducing medical expenditures associated with functional limitations, especially in women who were found to have significantly higher incremental costs in this sample of adults with diabetes.

An important strength of this study is the use of a large sample of nationally representative adults with diabetes. In addition, this study adds new evidence to a sparse body of literature addressing sex and gender disparities and assessing differences in medical expenditures for adults with a chronic disease that causes a substantial financial burden. Despite the strengths, however, there are limitations that must be mentioned. First, the cross-sectional design does not allow for the assessment of cause and effect relationship; therefore, no causal inferences can be made from the findings. Second, given the availability of specific covariates within the dataset, certain factors that might influence the results such as health status, health care utilization, attitudes and beliefs, and baseline functioning levels were not accounted for in the analyses. In addition, diabetes duration and type of diabetes were not included in the analyses. Expenditures associated with pregnancy were not accounted for in the analysis and may have contributed to the differences in expenditures between men and women. Given that these analyses included adults with diabetes, however, it is possible that most cases were of type 2 diabetes. Third, the findings of this study are based on self-reported data; therefore, the possibility for under- and/or over-reporting and estimating is possible.

### Implications for practice and/or policy

The findings of this study are important, as they provide new information on differences in diabetes-related costs between men and women and identify factors associated with increased costs in women. In this sample of adults with diabetes, women had significantly higher medical expenditures, which may be the result of increased functional limitation and disability. These findings confirm the need for efforts that prevent the onset of diabetes in individuals with prediabetes and those at higher risk of developing diabetes. It also demonstrates a need for (1) strategies that slow the progression of diabetes-related complications, (2) guidelines that focus on the clinical care of women with both diabetes and disability, and (3) programs that promote the long-term prevention of disability in diabetes.

Clinical treatment plans must address and include management strategies for functional limitations in women with diabetes. Future research is needed to better understand the direction and relationship between diabetes and disability, and to develop interventions that focus on determining best practices for reducing the burden of disability in women with diabetes. More research is needed to elucidate additional factors associated with higher health care costs in women with diabetes. Further, policies are needed that address disability and offer approaches for minimizing direct and indirect costs associated with diabetes and disability; this is particularly important given the potential benefits provided by the ACA for patients diagnosed with diabetes. Finally, women should be informed about physical limitations and disability as a possible complication of diabetes, educated on approaches to minimize disability burden, and provided disability-related resources to help improve overall quality of life.

## Conclusions

The results of this study are important and provide new information about the differences in medical expenditures for men and women with diabetes. In this nationally representative sample of adults with diabetes, women had significantly higher medical expenditures compared with men, a finding possibly due to increased functional limitation and disability. These results signify a strong need for understanding the impact of physical limitation on diabetes outcomes and related medical expenditures, particularly for women with diabetes. It is also important to recognize the existence of differences in medical expenditures between men and women with diabetes. In addition, policy recommendations and strategies for clinical management are needed to minimize costs and optimize care for women with diabetes. Given that the health status of women in society strongly influences the health of entire families (*i.e.*, men, women, and children), it is imperative that all measures are taken to ensure women receive affordable, quality health care and that barriers preventing optimal care are identified, addressed, and reduced to improve outcomes and overall quality of life.

## Authors' Contributions

J.S.W. and L.E.E. designed the study. L.E.E. acquired and analyzed the data. J.S.W. and L.E.E. developed the analyses, interpreted the data, and critically revised the article for important intellectual content. All authors approved the final article.

## Disclaimer

This work represents the views of the authors and has not been published previously except in abstract form. It is not under consideration for publication elsewhere and has been approved by all authors.

## Funding Information

Effort for this study was partially supported by the National Institute of Diabetes and Digestive and Kidney Diseases (K24DK093699, R01DK118038, R01DK120861, PI: L.E.E.).

## Abbreviations Used

ACA: Affordable Care Act
ADL: activities of daily living
CI: confidence interval
GLM: generalized linear model
HC: household component
IADL: instrumental activities of daily living
MEPS: Medical Expenditures Panel Survey
MSA: metropolitan statistical area
NHW: non-Hispanic white
